# Genomic insights into Yanbian cattle: Breed-specific selective sweeps identified by whole-genome sequencing

**DOI:** 10.1371/journal.pone.0331448

**Published:** 2025-09-15

**Authors:** Jihye Baek, Chun-Long Yan, Qingshan Gao, Seungwoo Son, Hak-kyo Lee, Donghyun Shin, Chang-Guo Yan

**Affiliations:** 1 Department of Animal Biotechnology, Jeonbuk National University, Jeonju, Republic of Korea; 2 College of Agriculture, Yanbian University, Yanji, Jilin, China; 3 Engineering Research Center of North-East Cold Region Beef Cattle Science and Technology Innovation, Ministry of Education, Yanbian University, Yanji, Jilin, China; 4 Department of Agricultural Convergence Technology, Jeonbuk National University, Jeonju, Republic of Korea; Chattogram Veterinary and Animal Sciences University, BANGLADESH

## Abstract

The Chinese Yanbian Yellow cattle are an indigenous East Asian breed, closely related to the Korean Hanwoo cattle, and presumably share the same origin. However, unlike Hanwoo, which has undergone approximately 40 years of intensive artificial selection, Yanbian cattle have remained relatively unselected, preserving diverse genetic characteristics. In this study, we used whole-genome sequencing data from 45 individuals to investigate the unique traits in Yanbian cattle. To identify selective sweep regions and compare the results depending on different methods, we applied three approaches: cross-population extended haplotype homozygosity (XP-EHH), cross-population composite likelihood ratio (XP-CLR), and population branch statistics (PBS) based on the fixation index. Our results highlight the *PEX14* gene and *SIRT6* gene which play a role in cold adaptation, showing high XP-CLR value with clear evidence of fixation. Notably, the genomic region containing *PEX14*, which is involved in the browning of white adipose tissue in response to cold exposure, exhibited reduced nucleotide diversity and low Tajima’s D value in Yanbian cattle. This suggests that natural selection has acted on this gene to facilitate cold adaptation. Furthermore, genomic regions with early fixation events were primarily associated with environmental adaptation, whereas more recent fixation events were related to economically important traits. These findings enhance our understanding of the genomic characteristics of Yanbian cattle and support their potential for environmental adaptation, providing valuable insights for future improvement efforts.

## Introduction

*Bos taurus* (taurine) and *Bos indicus* (zebu) domestic cattle are descended from wild *Bos primigenius* [1]. Ever since they were domesticated, cows have evolved under selection pressure to meet human needs. In the modern era, cattle are classified as beef cattle, bred for the production of meat, or dairy cattle, bred for the production of milk. Further, in certain countries, cows are regarded as sacred animals. In 2024, global bovine meat production reached 78.7 million tons, representing a 2.8% increase from the previous year (FAO, 2025). The primary driver of this growth has been the expansion of beef production in Brazil, Australia, China, European Union, and India. Meanwhile, China has been a key factor in the growth of beef demand in Asia, with its own output reaching 7.8 million tons, which represents a 5% annual increase. As reported by FAO, the beef yield increase in China can be attributed to the rising domestic demand and the continuous improvement of farm management [2].

There are currently 53 indigenous cattle breeds in China [3]. In particular, Yanbian cattle are one of Chinese beef breed primarily raised in Northeast China. This breed was originally used as a draft animal and is now mainly used as beef cattle. A previous study [4] suggested that Yanbian Yellow and Hanwoo cattle share the same ancestor, as the forebear of Yanbian Yellow came with Korean immigrants to Gando approximately one hundred years ago. Therefore, although cattle in the Yanbian region of northeastern China share genetic similarities with Korean cattle, it is possible that different genes may be preserved from the period in which the two breeds lived separately in different environments.

In Korea, Hanwoo cattle have been under strong selection pressure to improve the economic traits of this breed since 1980. As a result, Hanwoo can produce high-quality meat characterized by delicate and rich marbling score. Furthermore, previous research has shown that Hanwoo cattle are typically raised in environments where the maximum air temperature reaches 28.4°C, the maximum relative humidity is 91.4%, and the minimum and wind-chill temperatures are −4.0°C and −6.2°C, respectively [5]. In contrast, Yanbian cattle live under chilly temperatures and icy ground for half the year, and the minimum temperature is −37°C [6]. Thus, unlike Hanwoo, Yanbian cattle may harbor specific genes associated with the resilience and adaptability to extremely cold climate.

Understanding beef cattle at the whole-genome level has become increasingly important to support sustainable breeding strategies, uncover genetic history [7] and improve specific traits such as meat quality [8,9] and environmental adaptation [9]. In this context, several studies have examined genetic characteristics specific to Yanbian cattle. Choi et al. investigated selective sweep regions in Yanbian and Hanwoo cattle by applying ZHp scores based on single nucleotide polymorphism (SNP) data [4], and also analysed copy number variations related to marbling score [10]. More recently, Zhang et al. compared Yanbian cattle from Northeast China and N’dama cattle from West Africa to identify selection signatures associated with environmental adaptation [6]. Similarly, a recent genome-wide study compared SNPs between cattle from northern and southern China, using fixation index (*F*_*ST*_) value to detect breed-specific signals [11].

However, comprehensive selection scans involving Yanbian and commercial cattle—aimed at identifying both fixed and ongoing selective sweep regions—remain limited. In this study, we applied various methods, such as cross-population composite likelihood ratio (XP-CLR), cross-population extended haplotype homozygosity (XP-EHH), and population branch statistics (PBS) with *F*_*ST*_ value, to detect breed-specific selective sweeps in the Yanbian population. The XP-CLR method applies a likelihood ratio test (LRT) to identify selective sweep regions based on allele frequency differentiation between two population [12]. This method is well-suited for detecting older selective sweeps by leveraging allele frequency differentiation and linkage disequilibrium patterns between populations. For example, XP-CLR has been successfully used to identify genomic regions associated with environmental adaptation, including the acclimation of high altitudes in Ethiopian cattle (e.g., *MSRB3*, *MC1R*, and *ABCB10*) [13] and heat tolerance in Dehong cattle (e.g., *HSF1*, *PLCB1*, and *PLCB4*) [14].

However, XP-CLR may have limited power to detect recent selective events where alleles are nearing fixation. To complement this, we applied XP-EHH, which detects recent selection by comparing the decay of haplotype homozygosity from SNP [15]. This method is especially sensitive when one population exhibits long-range haplotype homozygosity while the other does not. XP-EHH has been used in previous studies to identify genomic regions associated with economic traits, such as meat quality and quantity in cattle, including Hanwoo and Angus [16]. *F*_*ST*_ captures the population differentiation between two breeds, while population branch statistics (PBS) using *F*_*ST*_ values can detect breed-specific selection signals. For example, PBS analysis has identified genes such as *IL12A* and *BOLA*, which are associated with resistance to African trypanosomiasis and tick infestation in Nigerian zebu cattle [17]. In our study, we applied the PBS approach to identify Yanbian cattle-specific genomic regions that may have undergone selection.

We particularly focused on comparing results of the three methods and identifying genes potentially involved in cold adaptation, considering the unique environmental conditions in which the Yanbian cattle are raised. The Yanbian-specific genes detected using various methods may contribute to our understanding of the differences between the Yanbian and Hanwoo breeds, and to breeding for greater adaptation to global warming.

## Materials and methods

### Data preparation, re-sequencing, and variant calling

In this study, we used whole-genome sequencing data consisting of 45 samples, including Hanwoo (N = 17), Angus (N = 10), Holstein (N = 10), and Yanbian yellow cattle (N = 8). All cattle whole-genome sequencing data were retrieved from previously published SRA datasets in National Center for Biotechnology Information. The BioProject accession numbers for each breed are provided in S12 Table in S1 File. The Illumina platform with a paired-end library generated whole-genome sequencing data. Prior to sequence mapping, the quality of the FASTQ file was evaluated using FastQC v0.11.9 [18], which performs a per-base sequence check. To remove potential adapter sequence reads, Trimmomatic v0.39 [19] was used with the following options: LEADING:3 TRAILING:3 SLIDINGWINDOW:4:15 and MINLEN:36. The cleaned paired-end sequence reads were mapped against the reference genome (ARS-UCD1.2) using the Burrows-Wheeler Aligner version 0.7.17 [20] with default settings. Additionally, Picard version 2.23.2 (“Picard Toolkit”) was used as an open-source software package (2019, Broad Institute, GitHub Repository. https://broadinstitute.github.io/picard/; Broad Institute) to identify duplicate reads using ‘MarkDuplicates’, and to fix mate-pair information, using ‘FixMateInformation’ command line. Furthermore, we recalibrated base quality score using the ApplyBQSR option in Genome Analysis Toolkit (GATK) v4.4.0.0 [21]. Following the recalibration of the data, variants were called using GATK HaplotypeCaller. The quality of SNPs was controlled with the ‘--hwe 10^−6^’, ‘--maf 0.05’ and ‘--max-missing 0.05’ options, and insertions and deletions were removed by VCFtools 0.1.17 [22]. Using SnpSift version 5.2, the number of breed-specific SNP was counted, applying Bonferroni correction (**p* *< 0.05).

### Population structure analysis

We performed principal component analysis (PCA) using genome-wide complex trait analysis (GCTA) v1.91.4 [23] on quality-controlled SNP data to assess breed population structure via eigenvector. The neighbor-joining tree, which illustrates the clustering state across all samples, was constructed after we calculated a p-distance matrix using VCF2Dis v1.44 (https://github.com/BGI-shenzhen/VCF2Dis), built it with PHYLIP v3.697 [24], and visualized using Figtree v1.4.0 [25]. The nucleotide diversity was calculated for autosomes using VCFtools in sliding window of 50 kb and 25 kb overlap to determine differences among breeds. Prior to the admixture analysis, we pruned variants in linkage equilibrium using Plink 1.9 [26], with ‘--indep-pairwise 50 10 0.1’, as described in the Admixture manual. This was followed by population structure analysis using Admixture version 1.3.0 [27] with the value of K, the assumed number of ancestral populations, increasing from two to three. To validate the admixture inferred from Admixture analysis, we conducted *f*_3_ statistical tests with ADMIXTOOLS (v.8.0.2) [28] to test for admixture versus shared ancestry in Yanbian breed.

### Exploring selective-sweep regions

To improve the accuracy of data for selective-sweep detection, we estimated haplotypes and predicted the genotypes using the Beagle program, 5.4 version [29]. The regions that underwent intensive selection were detected using three methods: XP-CLR, XP-EHH, PBS [15,30,31]. The XP-CLR score was calculated using XP-CLR version 1.0 [31], with the Angus, Hanwoo, and Holstein breeds used as reference groups to detect Yanbian-specific selective-sweep regions. Although these reference breeds have also been subjected to artificial selection, our aim was to identify genomic regions where Yanbian cattle exhibit stronger or more recent selective sweeps compared to these commercial breeds. Therefore, all detected signals represent relative selective differences between Yanbian and the reference breeds, rather than absolute evidence of selection when compared to an unselected population. The option of including non-overlapping 50-kb sliding windows, a maximum of 600 SNPs in each window, and a correlation level of 0.95 were selected to down-weight the contribution of SNPs to the XP-CLR score. The selscan v1.3.0 program [15] was used to calculate the XP-EHH value with the same population setting, after which all scores were standardized using the norm program implemented in selscan v1.3.0 for each 50-kb region. Our cutoff process was based on the maximum XP-EHH values. Calculation of the *F*_*ST*_ value, following the estimation of Weir and Cockerham [32], was performed using VCFtools with a sliding window size of 50 kb and 20-kb steps. Negative or missing values were excluded as they undefined or biologically meaningless [33]. The distance between two breeds was calculated according to equation (1), and the PBS were calculated using results of equation (1) to identify Yanbian breed-specific genetic differences using equation (2):


$$D_{A,B}=-\text{log}(\text{1}-F_{ST}(A,B))$$ (1)

$${PBS}_{4}=\;\frac{2D_{YB,Han}+\;D_{YB,Hol}+D_{YB,Ang}-D_{Han,Hol}-D_{Han,Ang}}{4}$$ (2)

The top 1% of the empirical distribution was used as a threshold for all results derived from the three methods to represent a breed-specific selective sweep. This cutoff was adopted to facilitate meaningful gene ontology (GO) enrichment analysis and to reduce the likelihood of term saturation.

### Gene annotation and gene ontology (GO) analysis

The specific and selected genomic regions were annotated to overlapping genes using gene annotations from Ensembl DB’s UCD1.2 gene annotation database (http://www.ensembl.org). To reinforce the evidence of selection with a statistical test, we applied Tajima’s D analysis using VCFtools, employing 10 kb windows for genes > 100 kb and 5 kb windows for genes < 100 kb. This was performed to assess whether regions showing strong XP-CLR signals and introgression patterns also deviated from neutral expectations. We further assessed nucleotide diversity in these annotated regions using overlapping window size of 50 kb and 25 kb (gene size > 100 kb) or 1 kb and 0.5 (gene size < 100 kb). Finally, the Database for Annotation, Visualization, and Integrated Discovery (DAVID) [34] was used for GO analysis, and each biological process (BP)-related GO term was based on the number of genes and *p*-value < 0.05.

## Results

### SNP identification from whole-genome sequencing data

The whole-genome sequencing data from 45 cattle‒comprising 17 Hanwoo, 8 Yanbian, 10 Angus, and 10 Holstein‒, yielded an average of 14.3X coverage. Subsequently, approximately 9.9 billion reads were produced and aligned to the reference genome (ARS-UCD1.2), with an average alignment rate of 86.95%. A detailed breakdown of individual sample alignment rates is provided in S1 Table in S1 File. Breed-specific mapping rates were 80.50% for Yanbian, 83.52% for Hanwoo, 92.10% for Angus, and 92.80% for Holstein.

The number of SNPs was reduced from 24,815,204 to 11,541,622 following quality control using VCFtools (S2 Table in S1 File). The number of breed-specific SNPs in Angus, Hanwoo, Holstein, and Yanbian breeds were 8,528 in Angus, 15,707 in Hanwoo, 9,941 in Holstein, and 128 in Yanbian cattle. The total number of these SNPs is 34,304, which accounts for 0.297% of the entire SNP set.

### Population structure analysis

**Fig 1:**
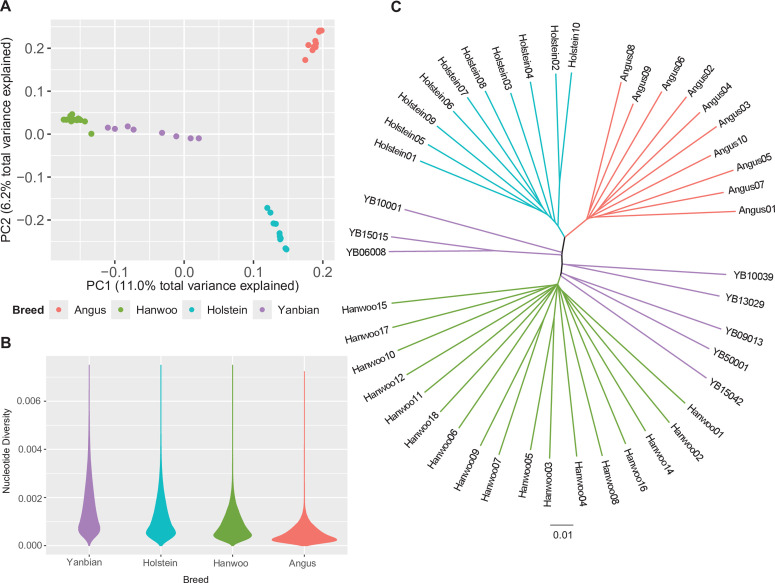
The visualization of relationship between the four breed populations. This illustration is following diagram: violet: Yanbian, green: Hanwoo, cyan: Holstein, and coral: Angus population. **(A)** Principal component analysis (PCA) of four cattle breeds (Angus, Hanwoo, Holstein, and Yanbian). **(B)** A violin plot to visualize the nucleotide diversity of each breed. **(C)** A neighbor-joining tree (NJ tree) of the four cattle breeds.

We performed PCA to evaluate the genetic position of a population composed of individuals (Fig 1A). The first and second principal components (PC1 and PC2) accounted for 11% and 6% of the total variance, respectively. European commercial breeds (Angus and Holstein) and Hanwoo cattle were clearly separated, while Yanbian cattle were positioned at an intermediate point along PC1. Along PC2, the two European breeds‒beef and dairy‒were distinguished according to their production purpose. However, the separation between Hanwoo and Yanbian samples in PC2 was not be clearly defined. PCA results also revealed intra-breed genetic diversity. For example, commercial breeds under intensive breeding system‒Hanwoo, Angus, and Holstein‒clustered closely along the PC1 axis. In contrast, Yanbian samples exhibited more widely dispersed, suggesting greater genetic variation compared to the commercial breeds. This finding differs from the patterns observed in other commercial breeds. We also observed notable differences in nucleotide diversity across breeds (Fig 1B). The violin plot of Yanbian cattle exhibited a longer and slimmer distribution compared to commercial breeds. Conversely, Angus, Hanwoo, and Holstein breeds showed more compressed violin shape, with Angus having the flattest profile. This indicates that the Yanbian samples used in this analysis possess greater diversity than the other breeds. Fig 1C represents a neighbor-joining tree illustrating genetic relationships among the four breeds. The tree revealed three clearly defined clusters and one group with ambiguous placement. The Western commercial groups formed one branch, which further split into Angus and Holstein. While the Hanwoo group spread evenly in the opposite direction, the Yanbian samples spread along an uneven branch, indicating a lack of unity among the samples. These non-clustering samples were located between the Hanwoo and Western European commercial groups, highlighting their intermediate genetic status.

**Fig 2:**
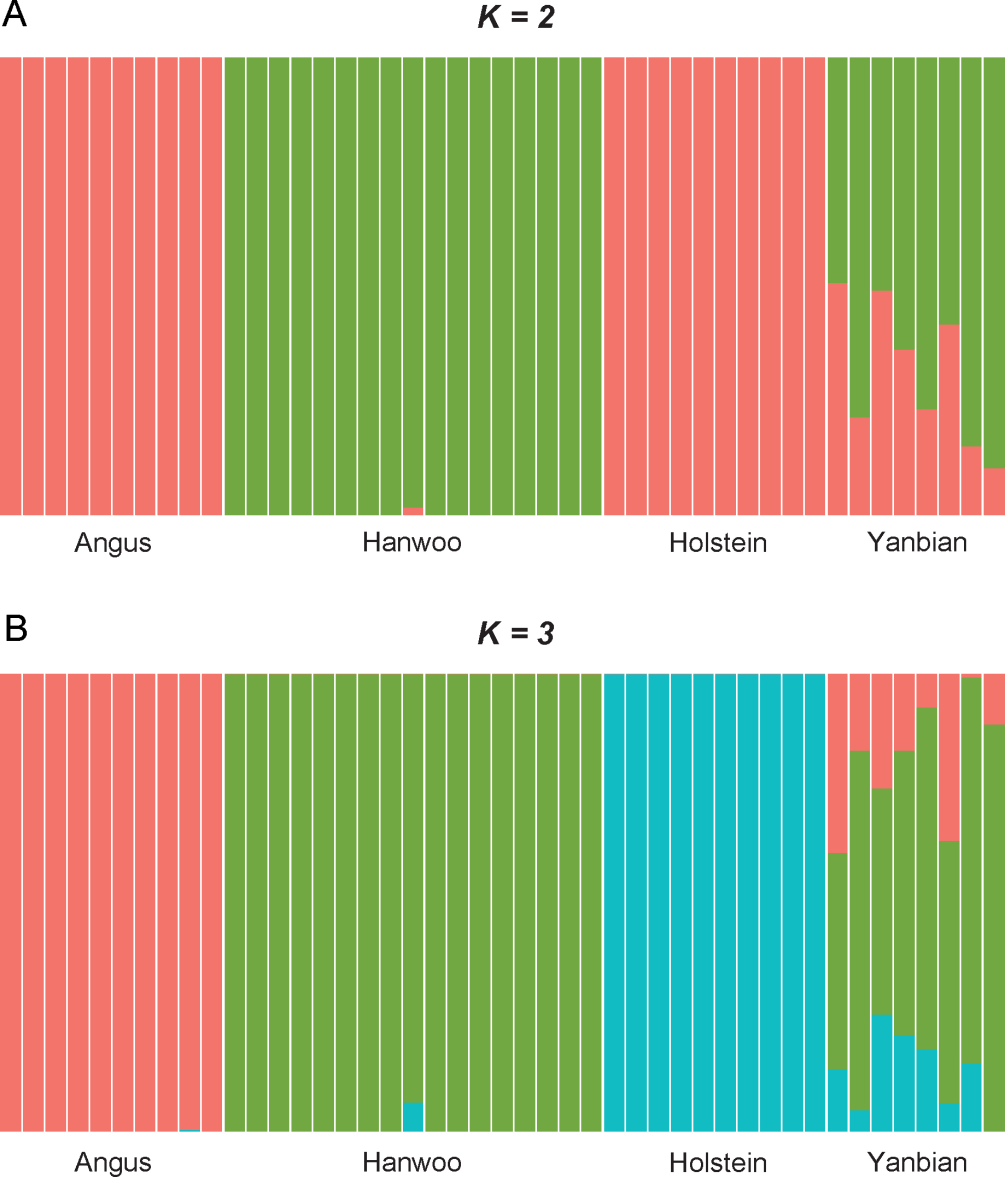
The inference of ancestry population across all samples. (A) and (B) present the results when K was set to 2 and 3, respectively. The varying proportions of color in each specimen indicated a differentiation in ancestral population composition with varying ancestry number.

We performed admixture analysis to infer the ancestral population structure among the four breeds. We tested K value of 2 and 3, and the results indicated that K = 3 best explained the population structure (Fig 2). At K = 2, a clear distinction was observed between European and Asian breeds (Fig 2A). However, the most Yanbian samples displayed admixture with Europe cattle. When K increased to 3, a distinct Holstein subpopulation emerged. At this level, Yanbian individuals exhibited admixture components from Hanwoo, Angus, and Holstein (Fig 2B). Although ADMIXTURE analysis suggested a mixture-like pattern in the Yanbian breed, *f*_3_ statistics provided no significant evidence of recent admixture (S3 Table in S1 File).

### Annotation and genetic validation of selective sweep signals

We identified selective-sweep regions using three population-based approaches (Table 1). To ascertain the composite likelihood ratio in the Yanbian population, three commercial breeds were alternatively selected as the reference model for the XP-CLR method. Thus, when the Angus breed was used as a reference population, 124 genes were detected (S1 Fig A in S1 File). The *FSD1* gene (XP-CLR = 373.91) had the highest XP-CLR value among these annotated genes. Furthermore, the peroxisomal biogenesis factor 14 (*PEX14*, XP-CLR = 294.79) and perilipin 4 (*PLIN4*, XP-CLR = 256.34) were also located within selective sweep reigons. In contrast, when the Hanwoo population was designated as the reference breed, the final numbers of selective regions and matched genes were 47,274 and 149, respectively. The *PLIN5* showed a selective signal of 155.26, and the *PEX14* gene (XP-CLR = 203.28) was also identified as observed previously (S1 Fig B in S1 File). Lastly, the use of Holstein as the reference breed yielded 476 selective-sweep regions, with 150 genes remaining after the application of the cutoff and removal of duplicate genes. Among these, *FSD1*, *SIRT6*, and *KCTD8* exhibited notably high XP-CLR values of 272.10, 177.17, and 125.85, respectively (S1 Fig C in S1 File).

**Table 1:** The number of the selective-sweep region detection analysis performed three methods.

Methods	Selected population	Reference population	Selective-sweep region	1% Empirical distribution	Annotated region^a^	Initial gene^b^
XP-CLR	Yanbian	Angus	47,579	476	176	124
		Hanwoo	47,274	473	193	149
		Holstein	47,584	476	194	150
**Methods**		**Reference population**	**Selective-sweep region**	**1% Empirical distribution**	**Annotated region** ^a^	**Initial gene** ^b^
XP-EHH	Yanbian	Angus	47,565	476	131	75
		Hanwoo	47,578	476	176	93
		Holstein	47,569	476	167	101
**Methods**			**Selective-sweep region**	**1% Empirical distribution**	**Annotated region** ^a^	**Initial gene** ^b^
Population branch statistics			62,668	672	323	148

^a^The number of matched regions with *Bos taurus* gene annotation information.

^b^The number of genes with duplicate gene regions removed.

In the XP-EHH method, which can detect haplotype homozygosity, the same breeds were used as reference populations. A total of 75 genes were identified as significant when Angus was used as the reference population. Three genes (*BSND, TMEM61,* and *GPATCH4*) located within the same sliding-window region, *Bos taurus* autosome (BTA) 3, had XP-EHH score exceeding 3.50. Additionally, the DNA polymerase epsilon 4 accessory subunit (*POLE4*) showed an XP-EHH value of 4.19. The *PLEKHF2* gene region (BTA 14:69174130–69194614) showed an XP-EHH value of 3.68 (S2 Fig A in S1 File). When the Hanwoo group was designated as the reference population, 476 significant regions and yielded 93 matching genes. Among these genes, kelch-like family member 38 (*KLHL38*, XP-EHH = 4.87) had the highest value, and the protein-coding gene Cornulin (*CRNN*, XP-EHH = 4.60) was assumed to be the result of selection (S2 Fig B in S1 File). In the case of the Holstein breed as a reference population, 101 genes were detected in the significant regions. The *KLHL38* gene showed significant XP-EHH values of 4.97 (S2 Fig C in S1 File).

Furthermore, to identify Yanbian-specific regions and genes, PBS analysis using *F*_*st*_ identified 148 genes derived from 627 regions. As shown in S3 Fig in S1 File, the potassium voltage-gated channel modifier subfamily S member 2 (*KCNS2*) gene region showed a notable PBS value of 0.28. In addition, *TRAM1L1* (PBS = 0.19) and *EDN3* (PBS = 0.21) showed considerable significance. We considered these regions as candidate gene, and a summary of the significant genes is presented in Table 2.

**Table 2:** The summary of Yanbian-specific candidate genes identified by three methods, with three different commercial breeds.

CHR^a^	Gene	Max XP-EHH^b^	XP-CLR^c^	PBS	Function	Reference population
16	*PEX14*	–	294.79	–	Cold acclimation	Angus and Hanwoo
7	*SIRT6*	–	177.17	–	Cold acclimation	Holstein
7	*FSD1*	–	373.91	–	Cold acclimation	Angus and Holstein
13	*EDN3*	–	–	0.20	Cold acclimation	–
7	*STAP2*	–	305.84	0.20	Carcass trait	Hanwoo
6	*STK32B*	–	176.75	–	Carcass trait	All XP-CLR
14	*PLEKHF2*	3.68	–	–	Carcass trait	Angus
14	*KLHL38*	4.97	–	–	Carcass trait	Holstein

^a^Chromosome.

^b^The maximum XP-EHH score within 50 kb sliding window, only the result with the highest value is pretended.

^c^Only the result with the highest value is pretended.

**Fig 3:**
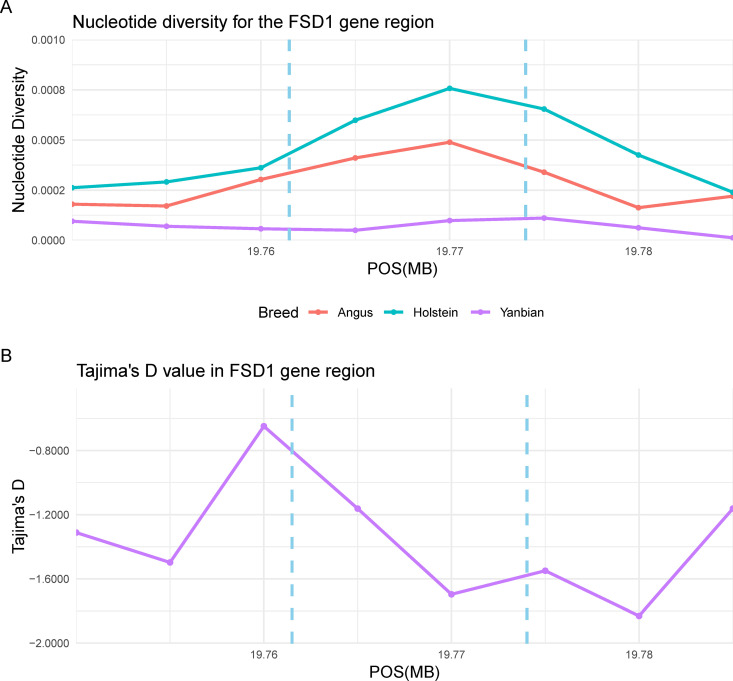
The line plot of nucleotide diversity and Tajima’s D around the *FSD1* gene (*Bos taurus* autosome 7: 19761502-19774023). (A) The violet, coral, and cyan lines indicated the nucleotide diversity of the Yanbian, Angus, Holstein breeds, respectively. The range of the *FSD1* gene was suggested by a sky blue vertical dashed line. (B) The Tajima’s D values around the *FSD1* gene regions of Yanbian breed.

**Fig 4:**
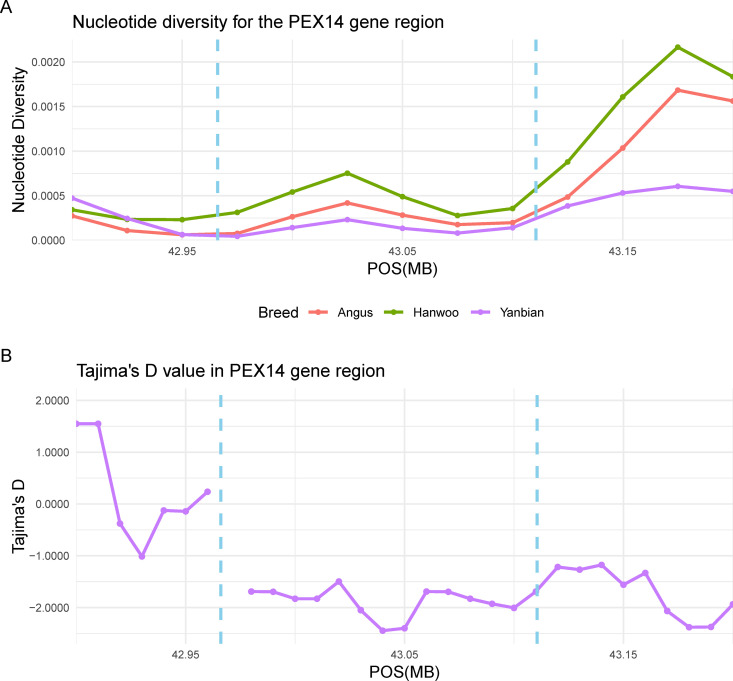
The line plot of nucleotide diversity and Tajima’s D around the *PEX14* gene (*Bos taurus* autosome 16: 42966075-43110580). (A) The violet, red, and green lines indicated the nucleotide diversity of the Yanbian, Angus, and Hanwoo breeds, respectively. The range of the *PEX14* gene was suggested by a sky blue vertical dashed line. (B) Tajima’s D values around the *PEX14* gene regions of Yanbian breed. Windows lacking SNPs are indicated by breaks in the line plot.

**Fig 5:**
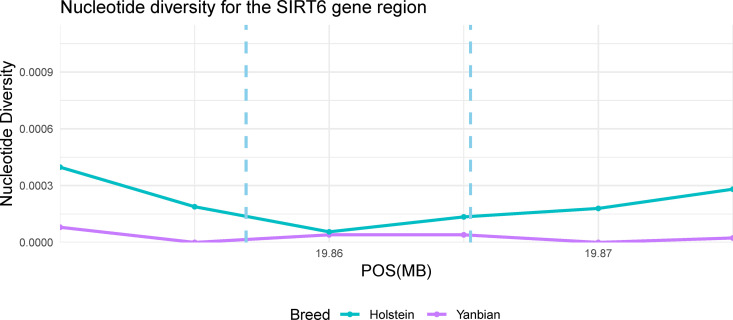
The Nucleotide diversity around the *SIRT6* gene (*Bos taurus* autosome 7: 19856911-19865253). The violet and cyan lines indicated the nucleotide diversity of Yanbian and Holstein breeds, respectively. The range of the *SIRT6* gene was suggested by a sky blue vertical dashed line.

To further examine the genetic patterns of these candidate gene regions, nucleotide diversity and Tajima’s D values were computed. The *FSD1* gene region showed significantly lower nucleotide diversity compared to other breeds (Fig 3A). In addition, Tajima’s D value sharply decreased around this gene region and were negative across the entire gene region (Fig 3B). As shown in Fig 4A, the results of *PEX14* gene region exhibited patterns similar to those of *FSD1* gene region. However, the disconnection in the Tajima’s D line plot around gene start site result from the absence of SNPs in this region (Fig 4B). Conversely, although the *SIRT6* gene showed low nucleotide diversity (Fig 5), Tajima’s D values were not obtained because too few SNPs were detected within the gene region (S4 Table in S1 File).

### Functional classification with gene ontology analysis

In the DAVID analysis, we selected the biological process classification to classify enriched biological processes among selective sweep genes. GO analysis using XP-CLR with Angus as the reference model revealed enrichment related to phosphorus metabolism (S5 Table in S1 File). A similar tendency was observed when the Holstein breed was used as a reference model (S7 Table in S1 File). Additionally, we highlighted nucleoside phosphate-related terms when the Hanwoo population was used as a reference model as highlighted in S6 Table in S1 File.

However, in XP-EHH analysis (S8 Table in S1 File), using Angus as the reference population demonstrated enrichment related to aminoacylation, nervous system, and pre-B cell allelic exclusion. When Hanwoo was used as the reference population (S9 Table in S1 File), nervous system-related terms were central, whereas using Holstein as the reference population was enriched for stimulus-related terms as illustrated in S10 Table in S1 File. Gene ontology analysis using the PBS method revealed that mitotic cytokinesis was a significant term (S11 Table in S1 File). In addition, seven of the ten terms were related to immunity.

## Discussion

### Genetic structural tendency in Yanbian breed

In this study, we used various analytical methods to identify the characteristics of Yanbian cattle that differ from those of commercial breeds. Foremost, the Yanbian samples analyzed showed higher nucleotide diversity than the other breed samples. This tendency can be directly explained and visualized, as shown in Fig 1B. Because of the higher degree of SNP diversity, the distance among samples was greater than that of other breeds in PC1 (Fig 1A). In the neighbor-joining tree (Fig 1C), Hanwoo, Angus, and Holstein samples originated from one root and formed a branch, whereas Yanbian samples showed a different aspect, with five roots and the distance separating them was relatively greater than that in the commercial breeds. These results indicated that the breeding of Yanbian cattle is still in progress. Artificial selection in Angus and Holstein breeds has had a long history of at least 100 years, whereas nationally intensive breeding of Hanwoo began only in 1979 in South Korea. We verified this interval as the difference in the number of copy number variations and the length of the runs of homozygosity reported in previous studies [35,36]. Contrary to the general current understanding, in this study, the diversity of Hanwoo cattle was slightly lower than that of Holstein cattle, although the difference was not significant, as estimated from the number of samples and sample selection. Furthermore, research based on microsatellite data has shown that Yanbian cattle have a higher degree of genetic diversity than Hanwoo or Holstein [37]. This previous report confirmed the reliability of our data and analytical results.

Secondly, we found that the genetic disposition of the Yanbian breed lies between that of the Hanwoo and European breeds. The results of the ancestry population inference (Fig 2) support this conclusion. In Fig 2A, we identified that the Yanbian samples shared ancestors not only with Hanwoo but also with common ancestors of European populations. The essence of these European commercial cattle was revealed to be the Angus and Holstein breeds in Fig 2B. However, as shown in S3 Table in S1 File, the admixture signal resulted not from introgression, but from shared genetic ancestry. In a previous study [38], the ancestry of the Yanbian sample consisted of Eurasian and East Asian cattle. The Hanwoo clearly belongs to the East Asian population, while Holstein belongs to the Eurasian population. However, the source of Angus’s ancestors in our analysis remains unclear and requires further research using a broader range of breeds. Taken together, these findings suggest that Yanbian cattle have undergone less intensive artificial selection, thereby preserving ancestral genetic variation that may contribute to environmental adaptability‒particularly to cold climates.

### Acclimatation to cold climate

Yanbian cattle have lived in cold climates for decades. Therefore, unlike commercial cattle breeds, we hypothesized that the selective-sweep regions identified in Yanbian samples would contain candidate genes related to cold climate adaptation. To explore the biological implications of the preserved regions, we investigated genes associated with cold exposure. Biologically, adipose tissue plays a role in energy storage, metabolic control, hormone secretion, insulation, and the protection of internal organs. There are two types of adipocytes: white adipose tissue (WAT) and brown adipose tissue (BAT). BAT arises from Myf5 + -expressing precursor cells to protect the body from exposure to cold by non-shivering thermogenesis via the differential expression of uncoupling protein 1 (UPC1) [39]. Therefore, we focused on the relationship between the selective signal regions and thermogenesis in BAT.

Foremost, we present the peroxisomal biogenesis factor 14 (*PEX14*) gene as evidence of adaptation to cold environments. This gene was located within the high XP-CLR region in both analyses using Angus and Hanwoo as reference breeds. As illustrated in S1 Fig in S1 File and Table 2, the XP-CLR value for this gene region was 294.79. The peroxisome, a membrane-bound organelle, has been investigated for its role in the destruction of reactive oxygen species by catalase. However, we focused on another function of peroxisomes in BAT. Ahlabo and Barnard demonstrated the presence of peroxisomes in rat BAT and established their role in thermogenesis [40]. The PEX14 protein is the principal element of peroxisomes, along with PEX13, and the mRNA expression of *PEX14* exhibits a marked increase during the non-shivering thermogenic process under cold exposure [41]. Similarly, WAT browning is induced by cold exposure and results in thermogenesis through diverse mechanisms, including hormone secretion and expression of genes such as UCP1 [42]. A study by Park et al. [41] reported that *PEX14* plays a role in WAT browning through its interaction with the PEX13 protein. The nucleotide diversity of the *PEX14* gene region (see Fig 4A) showed significantly low levels in the Yanbian group relative to the Angus and Hanwoo. As mentioned previously, although the nucleotide diversity of Yanbian was generally higher than commercial breeds (Fig 1B), the results of this gene region suggested that it was likely selected under selection pressures associated with cold adaptation. Furthermore, in this region, Tajima’s D values were consistently below −1 (Fig 4B), which is indicative of an excess of low-frequency polymorphisms. This pattern is characteristic of recent selective sweeps, suggesting that strong positive selection may have acted on these loci.

Our results highlight the significance of the *SIRT6* gene, which showed an XP-CLR value of 177.17 (Table 2 and S1 Fig C in S1 File), thus providing further evidence to support our discussion. The *SIRT6* region exhibited markedly low nucleotide diversity in Yanbian cattle, particularly within the exon region (Fig 5). However, in this gene region, Tajima’s D could not be calculated due to the near absence of polymorphic sites in Yanbian breed (S4 Table in S1 File). This lack of polymorphism may itself be indicative of selection signal, as it suggests that the genotype is largely fixed in this region due to strong selective pressure. A previous study [43] demonstrated that the SIRT6 protein level increased following cold exposure, and this protein is involved in the regulation of BAT thermogenesis and WAT browning in response to a cold challenge. Therefore, we concluded that the *SIRT6* gene might be a potential candidate for cold adaptation, given its role in regulating BAT thermogenesis and WAT browning upon cold exposure.

In addition, *PLIN5* (XP-CLR = 155.26), which was detected in a reference Hanwoo population, was investigated because of its importance in differentiating beige adipocytes in acute cold-treated mice and WAT browning activated by cold challenge [44,45]. In PBS, *EDN3* (PBS = 0.20) was considered to be related to the specific environment. The cold environment not only increased *EDN3*-mRNA expression and EDN3 protein levels but contributed to adipocyte browning as well by activating EDNRB signaling [46]. In the aforementioned context, monophosphate-activated protein kinase (AMPK) is upregulated in BAT and WAT of mice, during cold exposure [47]. Despite the fact that the AMPK signaling pathway was not directly enriched in the GO results, the phosphate metabolism-related terms were dominant in the XP-CLR results, including phosphate-containing compound metabolic process (GO:0006796). Given the critical role of AMPK in responding to cold exposure, these findings may reflect underlying metabolic adaptation that supports energy homeostasis under cold stress.

Notably, these genes were identified exclusively through the methods used herein, with the exception of XP-EHH, presumably because the selection signal in this region emerged earlier than in other detection methods, given that cold adaptation is essential for survival.

### Gene preservation of Yanbian cattle

The intensive breeding program for Yanbian cattle is still ongoing, in contrast to those of other commercial breeds. As mentioned above, the number of Yanbian-specific SNPs is considerably smaller compared to those in commercial breeds. In addition, the genetic distance among individuals within the Yanbian population was relatively large (Fig 1A), and its nucleotide diversity is high (Fig 1B). Taken together with evidence suggesting shared ancestry with Hanwoo and European breeds (Fig 2 and S4 Table in S1 File), these observations imply that the Yanbian breed has not undergone intensive selective breeding and may still retain a broad range of genetic variation. Therefore, we could trace the evolution of diverse ancestral variants in the Yanbian cattle genome. We detected Yanbian-specific genes that are advantageous carcass-associated traits by probing the selective-sweep region (Table 2). Further, the XP-CLR value of the *STAP2* gene was 305.84 when the Hanwoo breed was reference population (S1 Fig B in S1 File). This gene was classified as a relevant gene for the redness of the meat color of beef in a previous study [48]. The *STK32B* gene was selected because SNPs in this gene were associated with marbling score in Hanwoo cattle [49], and this gene was detected in all cases of XP-CLR. In turn, the *SIRT6* gene, which was previously inferred to be associated with cold adaptation, had a relationship with indigenous Chinese *Bos taurus* because of its unique carcass traits [50,51]. Thus, for example, backfat thickness and marbling score were reportedly higher when the *SIRT6* gene was highly expressed [52]. In previous GWAS results, which investigated carcass trait-associated genes in beef cattle, the GBLUP GWAS method was used to detect the *KCTD8* gene in the SNP window related to the rib-eye area [53]. In this study, this gene region was detected exclusively in the Holstein reference analysis results. These results confirmed that the XP-CLR method can highlight the beef-associated characteristics in Yanbian cattle when compared to Holstein cattle. Similarly, previous exploration of Hanwoo carcass traits using haplotype-based GWAS found that the *KLHL38* gene region was a significant component of the gene network associated with carcass weight gain [54]. This gene was also detected in our study when Hanwoo and Holstein populations were used as references (XP-EHH = 4.97), confirming the potential of Yanbian cattle as a valuable beef cattle resource.

Furthermore, we identified genes related to the Holstein breed and regions requiring further research. In particular, *BSND* (XP-EHH = 3.50) and *TMEM61* (XP-EHH = 3.50) genes were uniquely significant when the Angus breed was used as the reference population. These genes were investigated because of its association with the digital cushion trait in Holstein cattle in a previous report [55]. Furthermore, *POLE4* (XP-EHH = 4.19) is reportedly a candidate gene for lactation persistence in Holstein cows [56]. We speculated that this relationship with the Holstein breed arises from sharing ancestor. Despite the discussion on genes located in the selection signal region, there are still genes whose functions in cattle remain unresolved or ambiguous. For example, while the *FSD1* gene was identified as the most significant gene, it is relatively understudied in cattle. *FSD1*, a gene encoding a centrosomal protein, plays a role in anchoring microtubule asters to interphase centrosomes by binding to microtubules [57]. Microtubules are composed of α-tubulin and β-tubulin dimers, which tend to dissociate into monomers under cold stress [58]. Interestingly, considering *FSD1* gene regions showed high XP-CLR values (S1 Fig A and C in S1 File) and low nucleotide diversity and Tajima’s D (Fig 3), it may suggest that *FSD1* was under selection to repair or maintain microtubule‒centrosome connections disrupted by cold-induced microtubule deploymerization. However, further research is needed to verify this potential relationship. In particular, as the detection of this gene was limited to the European reference case, further research is needed to determine why it is a gene of high XP-CLR value compared to its status in European cattle. In another case, in this study, we identified *PLEKHF2* as a specific gene region that was detected only in the XP-EHH analysis of the Angus breed. According to Lee, this gene showed elevated mRNA expression in Hanwoo with low intramuscular fat [59]. We suggest that the detection in this region is attributable to the insufficient breeding process of beef cattle. In light of the above, there are multiple indications that Yanbian cattle breeding is progressing as beef cattle.

In this study, we investigated specific characteristics of the Yanbian breed using whole-genome sequencing. Although the sample size for the Yanbian breed population was relatively small (n = 8), it is comparable to those used in previous studies investigating selective sweeps in indigenous cattle breeds. Nevertheless, we acknowledge that the limited sample size may limit the statistical power of the analysis and affect the broader generalizability of the findings. Future studies with larger cohorts will be necessary to confirm and extend these findings. Despite the limited sample size, population structure analysis demonstrated that Yanbian cattle have undergone less intensive artificial selection compared to commercial breeds. Specifically, selective sweep region analyses suggest that the Yanbian cattle may possess genetic potential for both beef and milk production. While phenotypic data were unavailable in this study, incorporating genotype-phenotype associations in future research would help confirm the functional relevance of the candidate genes identified in this study. To support the interpretation of selective signals, we additionally examined the statistical properties of the candidate regions. While strong XP-CLR and XP-EHH signals are indicative of potential selection, we recognize that such patterns may also result from genetic drift, particularly in populations with small effective population sizes such as Yanbian breed. Although we calculated Tajima’s D values to further strengthen our inference, alternative explanations such as genetic drift or demographic effects may still remain and cannot be entirely excluded.

Collectively, genomic regions presumed to have undergone early fixation or to exhibit a high degree of population differentiation‒such as those identified through XP-CLR and PBS‒were primarily associated with environmental adaptation, likely reflecting the fact that such adaptations were essential for the survival of Yanbian cattle. In contrast, regions identified by XP-EHH, which tent to capture more recent fixation events, were generally related to economically important traits, suggesting that active selective breeding is currently underway in the Yanbian population. Our findings provide insights into the unique genetic landscape of Yanbian cattle and may inform future breeding strategies, particularly in the context of climate change.

## Supporting information

S1 File Supporting Information. S1 Table. Summary of 45 cattle sequencing results used in this analysis. S2 Table. The number of SNPs per chromosome. S3 Table. The results of *f*_3_ statistics. S4 Table. Tajima’s D value around *SIRT6* gene region of Yanbian cattle. S5 Table. The Gene ontology analysis top 10 terms which used the results of XP-CLR, Angus population was set reference model case. S6 Table. The Gene ontology analysis top 10 terms which used the results of XP-CLR, in case of the reference population was Hanwoo. S7 Table. The Gene ontology analysis top 10 terms which used the results of XP-CLR, when Holstein population was assumed reference population. S8 Table. The Gene ontology analysis top 10 terms which used the results of XP-EHH, Angus population was set reference population. S9 Table. The Gene ontology analysis top 10 terms which used the results of XP-EHH, the reference population was set Hanwoo breed. S10 Table. The Gene ontology analysis top 10 terms which used the results of XP-EHH, when Holstein population was reference population. S11 Table. The Gene ontology analysis top 10 terms which used the results of population branch statistics. S12 Table. The detailed information of used samples including SRA accession number and Bioproject number. S1 Fig. The Manhattan plot of the XP-CLR score distribution across all 29 autosomes of *Bos taurus*. The Yanbian breed was set as the selected population, with each of the three commercial breeds, Angus, Hanwoo, and Holstein, as a reference model in (A), (B), and (C), respectively. The x-axis indicates chromosome number, and the y-axis means XP-CLR value. The red horizontal line represents the empirical distribution of 1% thresholds. S2 Fig. The Manhattan plot of the distribution of XP-EHH scores on all 29 autosomes in *Bos taurus*. Each of the three commercial breeds, Angus, Hanwoo, and Holstein, as a reference population in (A), (B), and (C), respectively. The x-axis means chromosome number, and the y-axis indicates maximum XP-EHH value. The red horizontal line represents the empirical distribution 1% thresholds. S3 Fig. The Manhattan plot of population branch statistics results across all 29 autosomes of *Bos taurus*. The x-axis and y-axis indicate chromosome number and population branch statistics value, respectively. The red horizontal line represents the empirical distribution 1% thresholds.
(DOCX)
