# Supplementary material for: Genomic insights into Yanbian cattle: Breed-specific selective sweeps identified by whole-genome sequencing
Source: PLoS One. 2025 Sep 15;20(9):e0331448. doi: 10.1371/journal.pone.0331448 (PMC12435661; doi:10.1371/journal.pone.0331448)

**Supporting information**

**S1 Table. Summary of 45 cattle sequencing results used in this analysis**

| **Sample name** | **Breed** | **Raw reads** | **Mapped reads^a^** | **Properly paired reads^b^** | **Mapping rate^c^ (%)** |
| --- | --- | --- | --- | --- | --- |
| Angus01 | Angus | 201,448,342 | 189,982,039 | 186,997,650 | 92.83 |
| Angus02 | Angus | 210,744,870 | 198,358,112 | 195,120,040 | 92.59 |
| Angus03 | Angus | 189,887,544 | 173,631,391 | 171,169,692 | 90.14 |
| Angus04 | Angus | 191,272,048 | 175,394,968 | 172,824,480 | 90.36 |
| Angus05 | Angus | 209,556,896 | 190,085,735 | 187,656,600 | 89.55 |
| Angus06 | Angus | 428,308,644 | 396,611,107 | 391,620,394 | 91.43 |
| Angus07 | Angus | 513,396,908 | 468,258,297 | 457,707,374 | 89.15 |
| Angus08 | Angus | 527,367,042 | 475,877,361 | 468,339,650 | 88.81 |
| Angus09 | Angus | 480,938,978 | 443,404,240 | 438,017,620 | 91.08 |
| Angus10 | Angus | 395,540,374 | 365,612,914 | 358,996,586 | 90.76 |
| Hanwoo01 | Hanwoo | 409,263,992 | 373,478,558 | 331,764,260 | 81.06 |
| Hanwoo02 | Hanwoo | 406,157,756 | 368,626,156 | 318,580,158 | 78.44 |
| Hanwoo03 | Hanwoo | 429,134,000 | 385,495,820 | 339,561,216 | 79.13 |
| Hanwoo04 | Hanwoo | 394,033,908 | 360,668,023 | 327,389,894 | 83.09 |
| Hanwoo05 | Hanwoo | 427,523,498 | 389,581,061 | 366,174,594 | 85.65 |
| Hanwoo06 | Hanwoo | 385,376,588 | 350,780,776 | 315,506,300 | 81.87 |
| Hanwoo07 | Hanwoo | 393,763,586 | 358,759,388 | 315,583,098 | 80.15 |
| Hanwoo08 | Hanwoo | 353,765,412 | 326,530,352 | 295,514,598 | 83.53 |
| Hanwoo09 | Hanwoo | 352,530,756 | 323,558,321 | 288,606,900 | 81.87 |
| Hanwoo10 | Hanwoo | 367,021,022 | 336,792,495 | 310,479,572 | 84.59 |
| Hanwoo11 | Hanwoo | 435,224,260 | 272,436,156 | 267,742,012 | 61.52 |
| Hanwoo12 | Hanwoo | 374,506,710 | 236,673,067 | 232,860,976 | 62.18 |
| Hanwoo13 | Hanwoo | 435,853,968 | 335,930,018 | 329,974,370 | 75.71 |
| Hanwoo14 | Hanwoo | 425,061,722 | 353,486,451 | 347,564,536 | 81.77 |
| Hanwoo15 | Hanwoo | 492,959,082 | 385,712,586 | 374,652,844 | 76 |
| Hanwoo16 | Hanwoo | 407,435,410 | 313,849,055 | 308,420,228 | 75.7 |
| Hanwoo17 | Hanwoo | 438,697,180 | 289,745,659 | 285,348,180 | 65.04 |
| Holstein01 | Holstein | 374,831,012 | 349,260,662 | 336,089,644 | 89.66 |
| Holstein02 | Holstein | 358,246,600 | 336,613,795 | 331,752,756 | 92.6 |
| Holstein03 | Holstein | 295,552,724 | 277,424,004 | 265,924,724 | 89.98 |
| Holstein04 | Holstein | 699,074,820 | 644,639,284 | 633,986,892 | 90.69 |
| Holstein05 | Holstein | 387,849,368 | 353,333,903 | 346,970,892 | 89.46 |
| Holstein06 | Holstein | 535,374,584 | 479,413,204 | 469,127,094 | 87.63 |
| Holstein07 | Holstein | 486,733,876 | 454,978,111 | 445,936,700 | 91.62 |
| Holstein08 | Holstein | 490,076,188 | 456,360,014 | 443,573,470 | 90.51 |
| Holstein09 | Holstein | 483,313,372 | 452,448,188 | 442,642,900 | 91.59 |
| Holstein10 | Holstein | 483,087,754 | 453,582,502 | 444,653,870 | 92.04 |
| YB06008 | Yanbian | 582,735,270 | 462,703,011 | 449,681,746 | 77.17 |
| YB09013 | Yanbian | 575,924,356 | 463,919,440 | 449,356,688 | 78.02 |
| YB10001 | Yanbian | 523,981,766 | 427,767,382 | 411,428,184 | 78.52 |
| YB10039 | Yanbian | 652,862,110 | 520,946,606 | 505,928,212 | 77.49 |
| YB13029 | Yanbian | 853,981,668 | 686,646,824 | 668,088,270 | 78.23 |
| YB15015 | Yanbian | 619,907,992 | 512,966,324 | 496,164,296 | 80.04 |
| YB15042 | Yanbian | 649,874,510 | 518,064,697 | 499,170,632 | 76.81 |
| YB50001 | Yanbian | 591,843,470 | 471,555,792 | 460,524,916 | 77.81 |

^a^The number of mapped reads to the reference genome, ARS-UCD1.2. These reads filtered recalibration.

^b^The number of reads mapped to the correct direction

^c^The ratio of the number of properly paired reads to the total number of raw reads

**S2 Table. The number of SNPs per chromosome**

| **Chromosome** | **Number of SNPs** |
| --- | --- |
| 1 | 1,569,768 |
| 2 | 374,013 |
| 3 | 1,166,672 |
| 4 | 1,266,293 |
| 5 | 1,151,106 |
| 6 | 1,252,349 |
| 7 | 1,020,588 |
| 8 | 1,052,582 |
| 9 | 986,020 |
| 10 | 1,065,989 |
| 11 | 1,022,047 |
| 12 | 1,061,039 |
| 13 | 820,297 |
| 14 | 813,096 |
| 15 | 912,379 |
| 16 | 786,268 |
| 17 | 754,514 |
| 18 | 716,905 |
| 19 | 614,584 |
| 20 | 726,098 |
| 21 | 686,131 |
| 22 | 611,197 |
| 23 | 658,703 |
| 24 | 677,420 |
| 25 | 484,142 |
| 26 | 514,129 |
| 27 | 511,460 |
| 28 | 492,191 |
| 29 | 622,022 |
| MT | 946 |
| X | 424,256 |
| Total | 24,815,204 |

**S3 Table.** **The result of** $\boldsymbol{f}_{\boldsymbol{3}}$ **statistics**

| **Source 1** | **Source 2** | **Target** | **f_3** | **std.err** | **Z** |
| --- | --- | --- | --- | --- | --- |
| Hanwoo | Holstein | Yanbian | 0.014834 | 0.000709 | 20.92 |
| Hanwoo | Angus | Yanbian | 0.038587 | 0.000907 | 42.52 |
| Angus | Holstein | Yanbian | 0.058474 | 0.001067 | 54.791 |

**S4 Table. Tajima’s D value around *SIRT6* gene region of Yanbian cattle**

| **CHR** | **START** | **No. of SNPs** | **TajimaD** |
| --- | --- | --- | --- |
| 7 | 19,845,000 | 1 | -1.16221 |
| 7 | 19,850,000 | 2 | 0.837748 |
| 7 | 19,855,000 | 0 | NaN |
| 7 | 19,860,000 | 0 | NaN |
| 7 | 19,865,000 | 1 | 0.649981 |
| 7 | 19,870,000 | 0 | NaN |

**S5 Table. The Gene ontology analysis top 10 terms which used the results of XP-CLR, Angus population was set reference model case**

| **Term** | **Count** | ***p*-Value** | **Genes** | **Fold Enrichment** |
| --- | --- | --- | --- | --- |
| phosphate-containing compound metabolic process | 17 | 0.005 | RNASEH2B, MAP2K2, PPM1M, DGKB, IRAK3, ADCY2, ABHD5, FHIT, HK2, MTMR7, FER, SUCLA2, SPTLC2, EPHB1, STK32B, MAP3K5 | 2.149 |
| phosphorus metabolic process | 17 | 0.006 | RNASEH2B, MAP2K2, PPM1M, DGKB, IRAK3, ADCY2, ABHD5, FHIT, HK2, MTMR7, FER, SUCLA2, SPTLC2, EPHB1, STK32B, MAP3K5 | 2.113 |
| intracellular signal transduction | 15 | 0.006 | WWOX, MAP2K2, ARHGEF12, DGKB, IRAK3, ADCY2, PRR5L, FHIT, VAV2, NRG3, RARB, STK32B, RALGPS1, MAP3K5 | 2.250 |
| response to organonitrogen compound | 8 | 0.011 | GABRB2, SDK1, FER, CDH1, RARB, IRAK3, UBE4B, UBXN6 | 3.294 |
| positive regulation of metabolic process | 21 | 0.019 | WWOX, DYNC1H1, MAP2K2, KCNE2, PRDM15, IRAK3, PRR5L, ABHD5, LARP4B, EFNA5, ACTG2, PTMS, TOM1L1, RYBP, SPTLC2, CDH1, NSD1, RARB, ZFPM2, ARID2, SPTBN1 | 1.690 |
| cell-cell adhesion | 7 | 0.021 | SDK1, FER, CDH1, TLN2, CTNNA2, EFNA5, ASTN1 | 3.208 |
| regulation of protein phosphorylation | 8 | 0.024 | TOM1L1, IBTK, FER, CCNH, NSD1, IRAK3, PRR5L, EFNA5 | 2.797 |
| regulation of organelle organization | 10 | 0.026 | TOM1L1, CUL9, DYNC1H1, FER, MAP2K2, CDC23, FSD1, CTNNA2, EFNA5, SPTBN1 | 2.337 |
| synapse organization | 5 | 0.030 | GABRB2, SDK1, CDH1, EFNA5, EPHB1 | 4.246 |
| inner ear receptor cell differentiation | 3 | 0.031 | GABRB2, CLRN2, GSDME | 10.766 |

**S6 Table. The Gene ontology analysis top 10 terms which used the results of XP-CLR, in case of the reference population was Hanwoo**

| **Term** | **Count** | ***p*-Value** | **Genes** | **Fold Enrichment** |
| --- | --- | --- | --- | --- |
| nucleoside phosphate metabolic process | 10 | 0.001 | GARS1, DCAKD, ENTPD6, ADCY2, NME5, FHIT, HK2, AK8, SLC25A13 | 3.897 |
| nucleoside phosphate biosynthetic process | 7 | 0.001 | GARS1, DCAKD, ADCY2, NME5, AK8, SLC25A13 | 5.797 |
| nucleobase-containing small molecule metabolic process | 10 | 0.002 | GARS1, DCAKD, ENTPD6, ADCY2, NME5, FHIT, HK2, AK8, SLC25A13 | 3.464 |
| phosphate-containing compound metabolic process | 20 | 0.002 | TRIO, DAPK2, DCAKD, ENTPD6, ADCY2, NME5, CDC25C, PLA2G7, FHIT, HK2, AK8, STK10, ABR, GARS1, FER, ULK2, STK32B, ROS1, SLC25A13 | 2.113 |
| regulation of molecular function | 14 | 0.003 | FEM1A, CCNH, ACTN1, COMMD1, PEX14, VAV2, ABR, PDGFD, TRAF6, SPOCK1, TBC1D22A, ROS1, CTSB, PLIN5 | 2.616 |
| response to mitochondrial depolarisation | 3 | 0.003 | PRKN, SQSTM1, HK2 | 36.692 |
| phosphorus metabolic process | 20 | 0.003 | TRIO, DAPK2, DCAKD, ENTPD6, ADCY2, NME5, CDC25C, PLA2G7, FHIT, HK2, AK8, STK10, ABR, GARS1, FER, ULK2, STK32B, ROS1, SLC25A13 | 2.078 |
| negative regulation of reactive oxygen species metabolic process | 3 | 0.007 | PRKN, HK2, PLIN5 | 23.850 |
| nucleotide biosynthetic process | 6 | 0.007 | GARS1, DCAKD, ADCY2, NME5, SLC25A13 | 5.021 |
| negative regulation of cellular component organization | 9 | 0.008 | TOM1L1, PRKN, DYSF, TBCD, ULK2, SPOCK1, ARHGEF18, NAT10, CTNNA2 | 3.152 |

**S7 Table. The Gene ontology analysis top 10 terms which used the results of XP-CLR, when Holstein population was assumed reference population**

| **Term** | **Count** | ***p*-Value** | **Genes** | **Fold Enrichment** |
| --- | --- | --- | --- | --- |
| phosphate-containing compound metabolic process | 21 | 0.002 | PTPRS, TRIO, SGMS1, MTMR14, DAPK2, IRAK3, ADCY1, ABHD5, CLN8, CDC25C, FHIT, HK2, CDC25A, PPP2CA, DUSP10, GRK5, PDE4B, OGDH, STK32B, MET, MARK2 | 2.135 |
| phosphorus metabolic process | 21 | 0.002 | PTPRS, TRIO, SGMS1, MTMR14, DAPK2, IRAK3, ADCY1, ABHD5, CLN8, CDC25C, FHIT, HK2, CDC25A, PPP2CA, DUSP10, GRK5, PDE4B, OGDH, STK32B, MET, MARK2 | 2.100 |
| cell division | 8 | 0.004 | BABAM2, CDC23, ZBTB16, CHMP1A, SPIRE2, FSD1, CDC25C, CDC25A | 3.988 |
| vesicle-mediated transport | 16 | 0.004 | DENND1B, CUBN, RTN3, AP3B1, EPS15L1, COMMD1, SYT17, MYO1E, LRRC7, CHMP1A, SPIRE2, EXOC6, STX3, MET, VPS28, SH3GL1 | 2.269 |
| neurotransmitter transport | 5 | 0.005 | SLC6A6, SV2B, STX3, CLN8, SYT17 | 7.287 |
| regulation of protein ubiquitination | 5 | 0.006 | WDR48, TCF25, RASSF5, COMMD1, VPS28 | 6.893 |
| vesicle-mediated transport to the plasma membrane | 5 | 0.006 | DENND1B, LRRC7, EXOC6, COMMD1, STX3 | 6.831 |
| negative regulation of phosphorus metabolic process | 6 | 0.006 | HDAC4, PPP2CA, IBTK, DUSP10, IRAK3, PRR5L | 5.129 |
| negative regulation of phosphate metabolic process | 6 | 0.006 | HDAC4, PPP2CA, IBTK, DUSP10, IRAK3, PRR5L | 5.129 |
| intracellular signal transduction | 17 | 0.008 | HDAC4, WWOX, WSB2, DAPK2, IRAK3, PRR5L, ADCY1, PPP1R9A, FHIT, VAV2, BABAM2, DCDC2C, UIMC1, PDE4B, STK32B, RALGPS1, MARK2 | 2.052 |

**S8 Table. The Gene ontology analysis top 10 terms which used the results of XP-EHH, Angus population was set reference population**

| **Term** | **Count** | ***p*-Value** | **Genes** | **Fold Enrichment** |
| --- | --- | --- | --- | --- |
| nervous system process | 10 | 0.004 | OR8B1R, NPFF, OR5P86, OR8D6, NEFL, CRYBA1, TAAR5, VDAC1, OR5P1C, OR5P76B | 3.157 |
| histidyl-tRNA aminoacylation | 2 | 0.005 | HARS1, HARS2 | 357.053 |
| amino acid metabolic process | 5 | 0.006 | LRRC47, DHFR, HARS1, FAH, HARS2 | 6.866 |
| system process | 11 | 0.006 | OR8B1R, OXTR, NPFF, OR5P86, OR8D6, NEFL, CRYBA1, TAAR5, VDAC1, OR5P1C, OR5P76B | 2.675 |
| tRNA aminoacylation for protein translation | 3 | 0.006 | LRRC47, HARS1, HARS2 | 24.911 |
| tRNA aminoacylation | 3 | 0.007 | LRRC47, HARS1, HARS2 | 23.286 |
| amino acid activation | 3 | 0.007 | LRRC47, HARS1, HARS2 | 22.791 |
| pre-B cell allelic exclusion | 2 | 0.011 | RAG2, RAG1 | 178.526 |
| pre-B cell differentiation | 2 | 0.014 | RAG2, RAG1 | 142.821 |
| positive regulation of mitophagy | 2 | 0.022 | VDAC1, SLC25A4 | 89.263 |

**S9 Table. The Gene ontology analysis top 10 terms which used the results of XP-EHH, the reference population was set Hanwoo breed**

| **Term** | **Count** | ***p*-Value** | **Genes** | **Fold Enrichment** |
| --- | --- | --- | --- | --- |
| system process | 15 | 0.001 | OR9S36B, RCVRN, OR8B1R, GRXCR2, KCNJ11, LHFPL5, TAAR5, OR5P76B, OR9S39, HRH2, PIP, OR8D6, TLR9, NEFL, VDAC1 | 2.929 |
| nervous system process | 13 | 0.001 | OR9S36B, RCVRN, OR8B1R, GRXCR2, KCNJ11, LHFPL5, TAAR5, OR5P76B, OR9S39, PIP, OR8D6, NEFL, VDAC1 | 3.295 |
| sensory perception | 10 | 0.002 | OR9S36B, RCVRN, OR8B1R, GRXCR2, PIP, LHFPL5, OR8D6, TAAR5, OR5P76B, OR9S39 | 3.462 |
| positive regulation of NF-kappaB transcription factor activity | 3 | 0.010 | CRNN, TLR9, TICAM1 | 19.999 |
| positive regulation of cell development | 5 | 0.011 | CX3CR1, CHODL, TLR9, NEFL, TGM2 | 5.710 |
| positive regulation of neurogenesis | 4 | 0.012 | CX3CR1, CHODL, NEFL, TGM2 | 8.431 |
| sensory perception of chemical stimulus | 7 | 0.015 | OR9S36B, OR8B1R, PIP, OR8D6, TAAR5, OR5P76B, OR9S39 | 3.424 |
| response to molecule of bacterial origin | 4 | 0.021 | CX3CR1, NCR3LG1, TLR9, TICAM1 | 6.745 |
| positive regulation of DNA-binding transcription factor activity | 3 | 0.021 | CRNN, TLR9, TICAM1 | 13.230 |
| positive regulation of nervous system development | 4 | 0.021 | CX3CR1, CHODL, NEFL, TGM2 | 6.705 |

**S10 Table. The Gene ontology analysis top 10 terms which used the results of XP-EHH, when Holstein population was reference population**

| **Term** | **Count** | ***p*-Value** | **Genes** | **Fold Enrichment** |
| --- | --- | --- | --- | --- |
| phototransduction | 5 | 0.001 | RGR | 62.506 |
| detection of light stimulus | 5 | 0.001 | RGR | 40.445 |
| cellular response to light stimulus | 6 | 0.001 | RGR, COPS9 | 22.299 |
| cellular response to radiation | 6 | 0.001 | RGR, COPS9 | 16.838 |
| visual perception | 6 | 0.001 | CABP1, RGR | 14.226 |
| sensory perception of light stimulus | 6 | 0.001 | CABP1, RGR | 13.638 |
| detection of abiotic stimulus | 5 | 0.001 | RGR | 18.335 |
| detection of external stimulus | 5 | 0.001 | RGR | 18.094 |
| sensory perception | 12 | 0.001 | OR8B1R, BOTA-T2R10B, PIP, OR9S41, OR8D6, CABP1, RGR, OTOS | 3.986 |
| cellular response to abiotic stimulus | 6 | 0.001 | RGR, COPS9 | 9.707 |

**S11 Table. The Gene ontology analysis top 10 terms which used the results of population branch statistics**

| **Term** | **Count** | ***p*-Value** | **Genes** | **Fold Enrichment** |
| --- | --- | --- | --- | --- |
| mitotic cytokinetic process | 3 | 0.003 | CHMP1A, KLHDC8B, KIF20A | 36.343 |
| mitotic cytokinesis | 4 | 0.003 | CHMP1A, BIRC5, KLHDC8B, KIF20A | 13.216 |
| regulation of leukocyte activation | 8 | 0.004 | LAG3, CD4, TMIGD2, SPI1, PPP2R3C, SHPK, TICAM1, LAX1 | 3.887 |
| cell division | 7 | 0.007 | CDC23, CHMP1A, BIRC5, KLHDC8B, FSD1, KIF20A, CDC25A | 4.143 |
| organic hydroxy compound biosynthetic process | 5 | 0.007 | SREBF1, MC1R, CTNS, CYB5RL, IP6K1 | 6.584 |
| regulation of cell activation | 8 | 0.007 | LAG3, CD4, TMIGD2, SPI1, PPP2R3C, SHPK, TICAM1, LAX1 | 3.528 |
| cytoskeleton-dependent cytokinesis | 4 | 0.010 | CHMP1A, BIRC5, KLHDC8B, KIF20A | 9.086 |
| regulation of lymphocyte activation | 7 | 0.010 | LAG3, CD4, TMIGD2, SPI1, PPP2R3C, TICAM1, LAX1 | 3.831 |
| cytokinesis | 4 | 0.010 | CHMP1A, BIRC5, KLHDC8B, KIF20A | 8.864 |
| cytokinetic process | 3 | 0.011 | CHMP1A, KLHDC8B, KIF20A | 18.798 |

**S12 Table. The detailed information of used samples including Accession number and Bioproject number**

| **Sample ID** | **Accession number** | **BioProject number** |
| --- | --- | --- |
| Angus01 | SRR1525582 | PRJNA256210 |
| Angus02 | SRR1525583 | PRJNA256210 |
| Angus03 | SRR1525686 | PRJNA256210 |
| Angus04 | SRR1525687 | PRJNA256210 |
| Angus05 | SRR1525688 | PRJNA256210 |
| Angus06 | SRR1355237 | PRJNA176557 |
| Angus07 | SRR1365144 | PRJNA176557 |
| Angus08 | SRR1425124 | PRJNA176557 |
| Angus09 | SRR1365129 | PRJNA176557 |
| Angus10 | SRR1346376 | PRJNA176557 |
| Hanwoo01 | SRR934415 | PRJNA210519 |
| Hanwoo02 | SRR934417 | PRJNA210519 |
| Hanwoo03 | SRR934418 | PRJNA210519 |
| Hanwoo04 | SRR934419 | PRJNA210519 |
| Hanwoo05 | SRR934432 | PRJNA210519 |
| Hanwoo06 | SRR934433 | PRJNA210519 |
| Hanwoo07 | SRR934434 | PRJNA210519 |
| Hanwoo08 | SRR934435 | PRJNA210519 |
| Hanwoo09 | SRR934436 | PRJNA210519 |
| Hanwoo10 | SRR934437 | PRJNA210519 |
| Hanwoo11 | SRR934395 | PRJNA210523 |
| Hanwoo12 | SRR934397 | PRJNA210523 |
| Hanwoo13 | SRR934398 | PRJNA210523 |
| Hanwoo14 | SRR934400 | PRJNA210523 |
| Hanwoo15 | SRR934401 | PRJNA210523 |
| Hanwoo16 | SRR934402 | PRJNA210523 |
| Hanwoo17 | SRR934403 | PRJNA210523 |
| Holstein01 | SRR1346386 | PRJNA176557 |
| Holstein02 | SRR1346390 | PRJNA176557 |
| Holstein03 | SRR1346392 | PRJNA176557 |
| Holstein04 | SRR1348583 | PRJNA176557 |
| Holstein05 | SRR1348592 | PRJNA176557 |
| Holstein06 | SRR1365147 | PRJNA176557 |
| Holstein07 | SRR1425129 | PRJNA176557 |
| Holstein08 | SRR1425133 | PRJNA176557 |
| Holstein09 | SRR1425134 | PRJNA176557 |
| Holstein10 | SRR1425144 | PRJNA176557 |
| YB06008 | SRS9252157 | PRJNA737584 |
| YB09013 | SRS9252158 | PRJNA737584 |
| YB10001 | SRS9252159 | PRJNA737584 |
| YB10039 | SRS9252160 | PRJNA737584 |
| YB13029 | SRS9252161 | PRJNA737584 |
| YB15015 | SRS9252162 | PRJNA737584 |
| YB15042 | SRS9252163 | PRJNA737584 |
| YB50001 | SRS9252165 | PRJNA737584 |

**S1 Fig. The Manhattan plot of the XP-CLR score distribution across all 29 autosomes of *Bos taurus*.** The Yanbian breed was set as the selected population, with each of the three commercial breeds, Angus, Hanwoo, and Holstein, as a reference model in (A), (B), and (C), respectively. The x-axis indicates chromosome number, and the y-axis means XP-CLR value. The red horizontal line represents the empirical distribution of 1% thresholds.


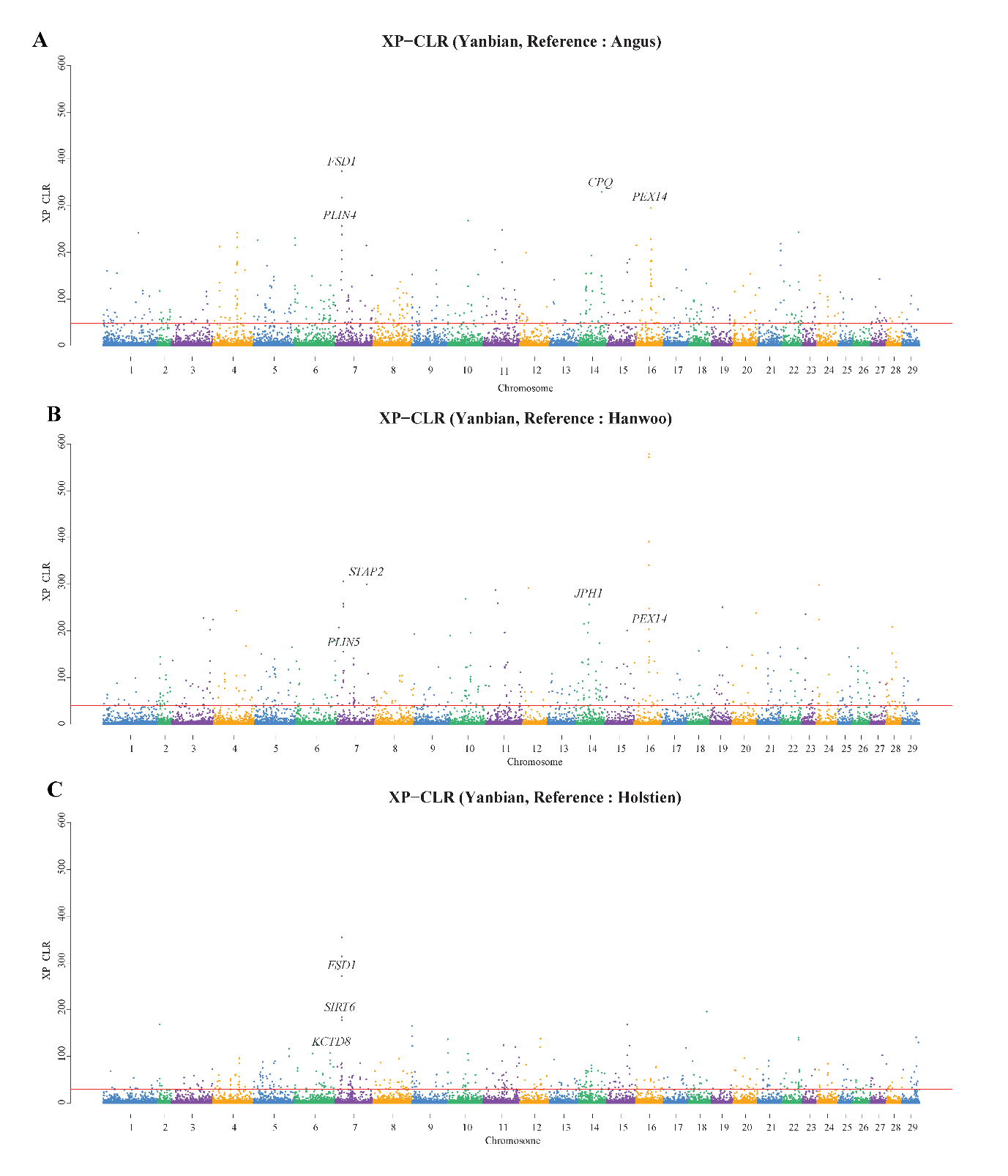


**S2 Fig. The Manhattan plot of the distribution of XP-EHH scores on all 29 autosomes in *Bos taurus*.** Each of the three commercial breeds, Angus, Hanwoo, and Holstein, as a reference population in (A), (B), and (C), respectively. The x-axis means chromosome number, and the y-axis indicates maximum XP-EHH value. The red horizontal line represents the empirical distribution 1% thresholds.


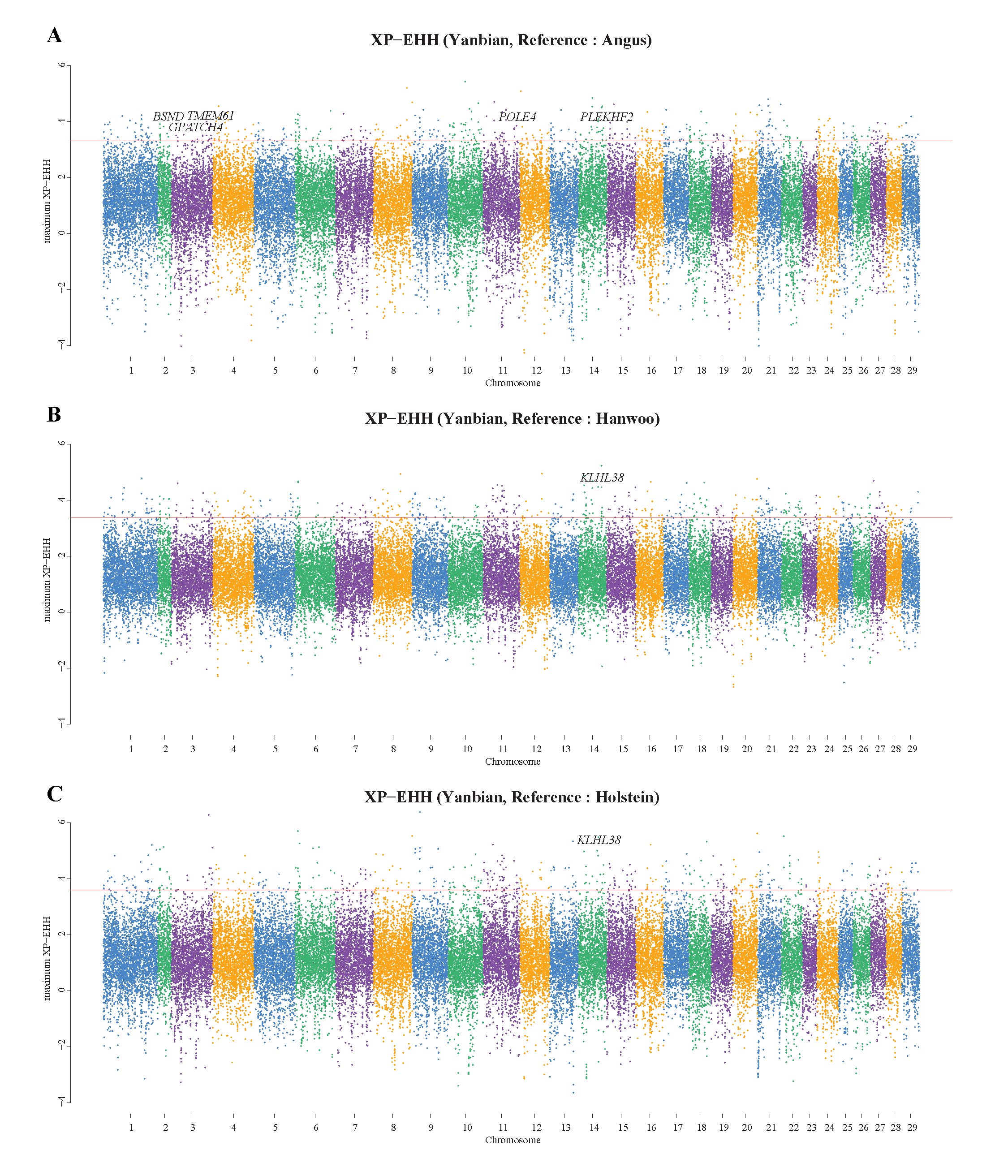


**S3 Fig. The Manhattan plot of population branch statistics results across all 29 autosomes of *Bos taurus*.** The x-axis and y-axis indicate chromosome number and population branch statistics value, respectively. The red horizontal line represents the empirical distribution 1% thresholds.


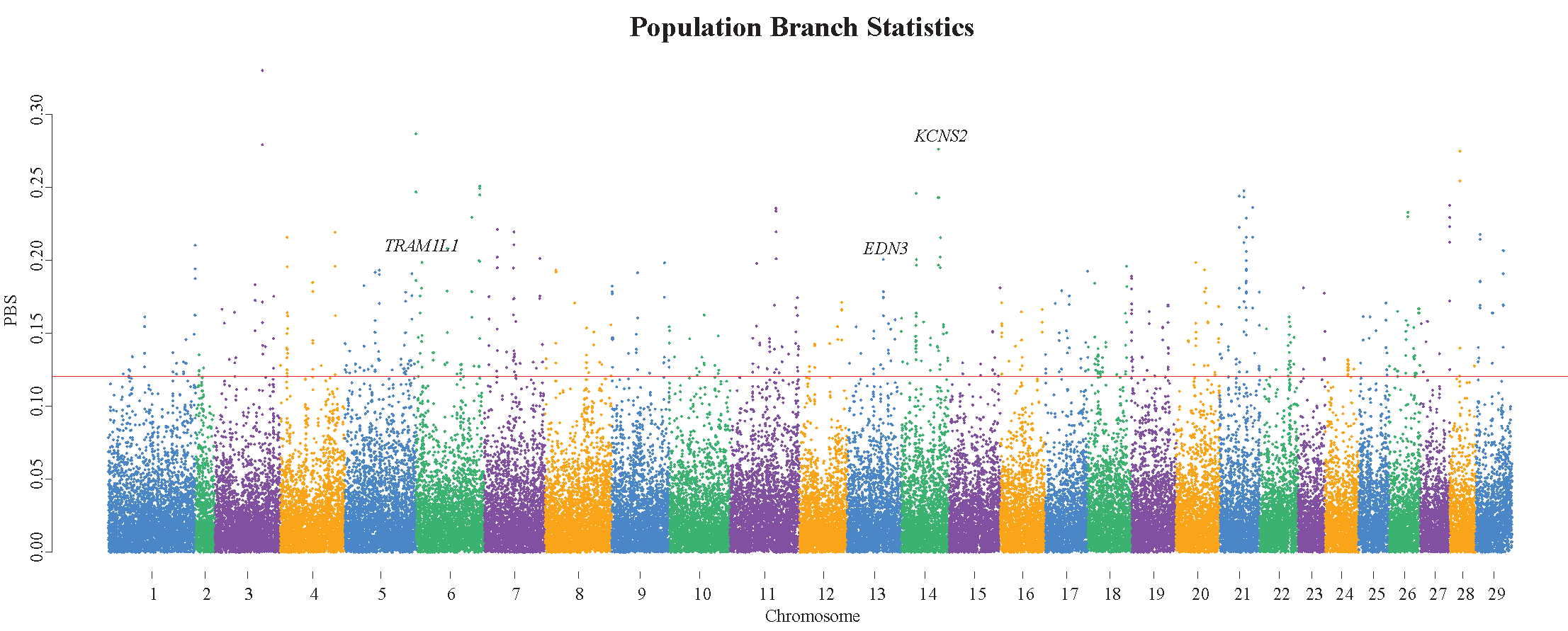

Supplement: S1 File — S1 Table. Summary of 45 cattle sequencing results used in this analysis. S2 Table. The number of SNPs per chromosome. S3 Table. The results of f3 statistics. S4 Table. Tajima’s D value around SIRT6 gene region of Yanbian cattle. S5 Table. The Gene ontology analysis top 10 terms which used the results of XP-CLR, Angus population was set reference model case. S6 Table. The Gene ontology analysis top 10 terms which used the results of XP-CLR, in case of the reference population was Hanwoo. S7 Table. The Gene ontology analysis top 10 terms which used the results of XP-CLR, when Holstein population was assumed reference population. S8 Table. The Gene ontology analysis top 10 terms which used the results of XP-EHH, Angus population was set reference population. S9 Table. The Gene ontology analysis top 10 terms which used the results of XP-EHH, the reference population was set Hanwoo breed. S10 Table. The Gene ontology analysis top 10 terms which used the results of XP-EHH, when Holstein population was reference population. S11 Table. The Gene ontology analysis top 10 terms which used the results of population branch statistics. S12 Table. The detailed information of used samples including SRA accession number and Bioproject number. S1 Fig. The Manhattan plot of the XP-CLR score distribution across all 29 autosomes of Bos taurus. The Yanbian breed was set as the selected population, with each of the three commercial breeds, Angus, Hanwoo, and Holstein, as a reference model in (A), (B), and (C), respectively. The x-axis indicates chromosome number, and the y-axis means XP-CLR value. The red horizontal line represents the empirical distribution of 1% thresholds. S2 Fig. The Manhattan plot of the distribution of XP-EHH scores on all 29 autosomes in Bos taurus. Each of the three commercial breeds, Angus, Hanwoo, and Holstein, as a reference population in (A), (B), and (C), respectively. The x-axis means chromosome number, and the y-axis indicates maximum XP-EHH value. The red [file pone.0331448.s001.docx]
